# Mechanisms of Regulatory B cell Function in Autoimmune and Inflammatory Diseases beyond IL-10

**DOI:** 10.3390/jcm6010012

**Published:** 2017-01-23

**Authors:** Avijit Ray, Bonnie N. Dittel

**Affiliations:** 1 Blood Center of Wisconsin, Blood Research Institute, Milwaukee, WI 53226, USA; rayavijit@gmail.com; 2 Oncology Discovery, AbbVie Inc., North Chicago, IL 60064, USA; 3 Department of Microbiology and Molecular Genetics, Medical College of Wisconsin, Milwaukee, WI 53226, USA

**Keywords:** B cell, immune regulation, autoimmunity, inflammation

## Abstract

In the past two decades it has become clear that in addition to antigen presentation and antibody production B cells play prominent roles in immune regulation. While B cell-derived IL-10 has garnered much attention, B cells also effectively regulate inflammation by a variety of IL-10-independent mechanisms. B cell regulation has been studied in both autoimmune and inflammatory diseases. While collectively called regulatory B cells (Breg), no definitive phenotype has emerged for B cells with regulatory potential. This has made their study challenging and thus unique B cell regulatory mechanisms have emerged in a disease-dependent manner. Thus to harness the therapeutic potential of Breg, further studies are needed to understand how they emerge and are induced to evoke their regulatory activities.

## 1. Introduction

Although B cells play a crucial role in mounting an immune response, they also possess immune-regulatory properties that have been elucidated extensively in the last decade [1]. Numerous studies have documented resolution of immune responses by varied subsets of B cells collectively referred to as regulatory B cells (Breg) [1]. Interestingly, Breg come in a variety of flavors differing in their phenotype, location and functional modality [2]. Although most studies have been focused on IL-10-mediated regulation by Breg, which have been extensively reviewed [3,4], other mechanisms including but not limited to GITRL-mediated regulatory T cell (Treg) maintenance, IL-35 and TGF-β production and adenosine generation via CD73 have also been well documented [5]. In this review, we will discuss IL-10-independent mechanisms of Breg function in the context of autoimmune and inflammatory diseases (Table 1).

## 2. Experimental Autoimmune Encephalomyelitis (EAE)

Using the EAE mouse model of the central nervous system autoimmune disease multiple sclerosis (MS), we provided the first evidence that B cells play a regulatory role in autoimmunity [24]. Specifically, we showed that B cell-deficient B10.PL mice immunized with the immunodominant myelin basic protein peptide Ac1-11 were unable to recover from the signs of EAE [24]. This seminal finding was confirmed using a similar strategy in C57BL/6 mice, which indicated a role for IL-10 in B cell regulation [25]. We also reproduced these results in the adoptive transfer model of EAE in B10.PL mice [26]. Given that B cell depletion in humans using antibodies specific for CD20 (rituximab) has clinical relevance in relapsing and remitting MS [27,28], we utilized the same strategy and found that B cell depletion prior to the onset of EAE also resulted in chronic EAE induced by adoptive transfer in B10.PL mice [6]. This same result was obtained in the MOG_35–55_ model of EAE in C57BL/6 mice [29,30]. Interestingly, B cell depletion during EAE progression suppressed disease severity, suggesting that B cells can also play a pathogenic role in EAE [29,31]. Mechanisms contributing to B cell pathogenesis demonstrated experimentally in EAE include antigen presentation and IL-6 production [31,32]. However, the absence of B cells did not alter the EAE disease course in relapsing and remitting EAE in (B10/PLxSJL/J)F_1_ mice [33]. These collective data indicate that B cells are not required for the initiation of disease, but likely play a role in progression of disease by a combination of antigen presentation and proinflammatory cytokine production. To date no definitive Breg markers have been identified, so determining when they emerge and where they exert their regulation has not been possible.

In subsequent studies, we have extensively studied the role of B cells in the context of EAE and were one of the first to identify an IL-10-independent mechanism of action of Breg [6]. In addition, we found that Breg regulation during EAE was also independent of antigen presentation [6]. Our investigations identified B cell involvement in the peripheral maintenance of Foxp3^+^ regulatory T cells (Treg), as genetic or antibody-mediated (anti-CD20) ablation of B cells resulted in significantly reduced numbers of Tregs in the periphery, without affecting their development in the thymus [6]. The reduction in Treg was correlated with the inability to resolve EAE leading to chronic disease [6]. Further investigation revealed a role for B cells in inducing Treg proliferation thereby contributing to their peripheral homeostasis via glucocorticoid-induced tumor necrosis factor-related receptor (GITR)-ligand (GITRL) [6]. Antibody-mediated blocking of GITRL on B cells abolished their Treg maintenance resulting in chronic EAE [6]. Further evidence supporting a role for GITRL expression by B cells driving Treg proliferation is the finding that transgenic mice over expressing GITRL in B cells have increased numbers of Treg [34]. A finding we recently confirmed in our animal colony using the same mice (Gurski and Dittel, unpublished observations).

Of interest, we found that B cell-derived IL-10 was not required for their ability to induce Treg proliferation [6]. Similarly, it was found that CD19^+^CD23^+^ Breg induced by Helminths, ameliorated EAE independent of IL-10 [7]. Recently, a mechanism of Breg function involving programmed death 1 (PD-1) and its ligand PD-L1 has also been observed [8]. It was found that adoptive transfer of PD-L1^Hi^ B cells resolved EAE by negatively regulating T cell differentiation [10]. Interestingly, PD-1 is highly expressed on Treg [35] and thus modulation of Treg by PD-L1^Hi^ B cells is a possibility. Indeed, it was shown that estrogen treatment, that resolves EAE [36], upregulated PD-1 on Treg [9], which was dependent on PD-L1 expression on B cells [8]. Apart from costimulatory molecules, recently it has been shown that Breg also utilize the cytokine IL-35 in regulating immune response in the context of EAE, although this mechanism is not independent of IL-10 [11]. B cells required both cytokines for resolving EAE as deficiency in either IL-35 or IL-10 abrogated the regulatory potential of B cells [11]. Mainly, the CD138^Hi^ antibody-secreting cells displayed this regulatory property via secretion of both IL-10 and IL-35 and they also dampened host immunity against infectious disease [11]. In a separate study, it was shown that B cells produce IL-35 in response to IL-35 stimulation and can control experimental uveitis [37]. Interestingly, Treg also produce IL-35 [38] and thus Treg-mediated generation of IL-35-producing Breg is a possibility suggesting cooperation between these two regulatory cell populations, as also observed by us [6].

The above collective data indicate that B cells play dual roles in EAE both in resolving CNS-directed autoimmunity and in promoting disease progression. Even though anti-CD20 depletion with rituximab has clinical relevance in MS, the exact mechanism(s) whereby B cell depletion is therapeutic in MS is not known. One mechanism that has largely been eliminated is a role for pathogenic antibodies, given that rituximab does not deplete plasma cells due their lack of CD20 expression. It is conceivable that pathogenic B cells are depleted thereby limiting subsequent relapses due to reduced antigen presentation to myelin-specific T cells or the production of proinflammatory cytokines. On the other hand, rituximab may either spare Breg subsets or that they are the first to emerge from the bone marrow following B cell depletion thus becoming the dominant B cell population, a mechanism that would also limit inflammation and subsequent relapses. While rituximab can reduce B cell numbers nearly to zero in the peripheral blood this does not occur in all patients [39]. In addition, based on mouse studies using B cell depletion with anti-CD20, depending on the isotype of antibody used, splenic B cells populations are not completely depleted [40]. Thus, we propose that Breg subsets are spared in the spleens of rituximab-treated MS patients where they maintain Treg cell numbers that in turn suppress T cell activation leading to relapses in MS. These data are consistent with the finding that Treg numbers are not altered following rituximab administration [41].

## 3. Type 1 Diabetes (T1D)

The dual role of B cells is very apparent in Type 1 diabetes (T1D) where insulin-producing B cells in the pancreas are targeted and damaged by self-reactive immune cells. In nonobese diabetic (NOD) mice, a model for T1D, although B cells initiate the autoimmune response via production of self-reactive antibodies, they also display a regulatory role. Activation via lipopolysaccharide (LPS), induced FasL expression and TGF-β secretion by B cells, which when adoptively transferred to NOD mice prevented the onset of disease [12]. Mechanistically, the activated B cells induced FasL-mediated apoptosis of self-reactive T cells and dampened the function of antigen presenting cells via TGF-β [12]. However, B cell depletion therapies have shown positive outcomes in NOD mice [42] and human trials [43], suggesting that the pathogenic role of B cells outweighs their regulatory potential in T1D.

## 4. Colitis

In a spontaneous enterocolitis model using TCRα^−/−^ mice, absence of B cells exacerbated disease [44] and the mechanism of Breg function was attributed to natural IgM production by B1 cells in response to commensals [13]. Although B1 cells are potent producers of IL-10 [45], in this study, there was no detectable change in IL-10 production by B cells either exposed to commensals or not. Commensal mediated IgM production by B cells was also implicated in Breg function in a model of colonic inflammation induced by dextran sodium sulfate (DSS) [14]. Apart from commensals, *Hymenolepis diminuta* infection also generated Breg that could control DSS-colitis probably via TGF-β-mediated regulation of inflammatory macrophages [15]. Also, CD73-mediated generation of adenosine has been implicated in the regulatory function of B1 B cell in DSS-colitis [16]. Interestingly, it was found that adenosine production was reduced when B cells were deficient in IL-10 [16], although whether IL-10-mediated regulation of CD73 expression occurs is not known. Interestingly, Treg express the adenosine receptor [46,47], which could be activated by adenosine generated by Breg. This suggests interplay between Breg and Treg for controlling an immune response, which was also observed by us in DSS-colitis [17]. Specifically, we observed a regulatory role for B cells in controlling DSS-colitis via their interaction with Treg which in turn induced IgA production by B cells [17].

## 5. Transplant Tolerance

In a cardiac allograft model, anti-CD45RB treatment prevented graft rejection, which was shown to be dependent on the presence of B cells [48]. B cell deficiency ameliorated the efficacy of anti-CD45RB therapy in promoting graft tolerance, which was restored by reconstituting the host with B cells via adoptive transfer [48]. Anti-CD45RB treatment upregulated ICAM-1 on B cells that was required for the immune regulation by B cells [18] whilst IL-10 production was indispensable [49]. Further investigation revealed TGF-β-mediated expansion of Treg as the mechanism of Breg function in this model [19]. Similarly, in a MHC I mismatch skin graft model, transitional-2 B cells from tolerized mice were immunosuppressive and prolonged graft survival upon adoptive transfer, even when deficient in IL-10 production [20]. In another long-term cardiac allograft model, FcγRIIB^Hi^ B cells accumulated over time and were involved in immune regulation preventing graft rejection (47). In addition, in a male-to-female skin graft model FasL^+^ B cells mediated immune tolerance resulting in acceptance of male skin grafts by female recipients [22]. Deficiency in FasL on B cells abrogated their tolerogenic potential [22]. Although not investigated, a probable mechanism is B cell-mediated apoptosis of Fas expressing effector T cells via Fas-FasL interactions, a mechanism also observed in an animal model of rheumatoid arthritis (RA), collagen-induced arthritis (CIA).

## 6. CIA

In CIA, B cell-mediated immune regulation through induction of apoptosis in pathogenic T cells via Fas-FasL is suggested [23]. In this model increased disease severity was correlated with reduced presence of FasL^+^CD5^+^ B cells and decreased T cell death [23]. Interestingly, CD5^+^ B1 cells are also potent producers of IL-10 [45] and in this study dependency of Breg on this cytokine was not evaluated. Thus a possibility exists for codependence of Breg function on both FasL expression and IL-10 production, as with IL-35-producing Bregs in EAE [11].

## 7. Conclusions

Although a regulatory role for B cells is well established, much needs to be elucidated regarding their origin, phenotype and function. The variety in phenotype, mechanism of action, and location of B cells with suppressive ability suggest that unlike Treg, Breg are not a distinct lineage. Rather, depending on the context, B cells can either, transiently or permanently, display regulatory potential for suppressing an immune response and prevent immune pathology. Thus, it is important to identify the cues that lead to the generation of Breg to facilitate the therapeutically harnessing the potential of these cells in controlling immune responses.

## Figures and Tables

**Table 1:** Regulatory B cell mechanisms independent of IL-10.

Disease	Breg Phenotype	Mechanism of Action	Refs.
**EAE**	Splenic	GITRL-mediated homeostatic maintenance of Treg	[6]
	CD19^+^CD23^Hi^ MLN	Unknown	[7]
	PD-L1^+^	Upregulation of PD-1 on Treg	[8,9]
	PD-L1^Hi^	Restriction of T cell differentiation	[10]
	IL-35-producing	IL-35 production	[11]
**Diabetes**	LPS-activated	FasL-mediated apoptosis of pathogenic T cells and TGF-β	[12]
**Colitis**	B1	Natural IgM	[13,14]
	Splenic	Probably via TGF-β-mediated regulation of inflammatory macrophages	[15]
	B1	CD73 generation of adenosine	[16]
	Unknown	Treg induced production of IgA	[17]
**Transplant tolerance**	ICAM-1^Hi^	TGF-β mediated expansion of Treg	[18,19]
	Transitional-2	Unknown	[20]
	FcγRIIB^Hi^	Unknown	[21]
	FasL^+^	Possibly Fas: FasL-mediated killing of effector T cell	[22]
**CIA**	FasL^+^CD5^+^	T cell apoptosis via Fas: FasL	[23]
